# Infants Eating and Sleeping

**Published:** 1860-12

**Authors:** 


### INFANTS EATING AND SLEEPING.

IN our new book on SLEEP, as to the importance of sleeping soundly and in a pure atmosphere, the following suggestions are for the abatement of the nuisance of crying and colicky babies, for how can a poor fellow, who has been working hard all day in brain or body, get any kind of rest, repose, and recuperation, when there is a little responsibility a yawping and a squirming around, as if a young boa-constrictor were experimenting on every bone of its body! We would just like to know how it is possible to get even a scintillation of a doze, under the very afflicting and inflicting circumstances of the case. Now for the remedy, at least in part.

An infant should not be allowed to sleep for several hours previous to its bed-time, which should be about one hour after sun-down, when it should be fed and put to sleep. When the mother retires, it should be fed again; then if the crib be on the same level with the bed, and close to it with the side let down, the mother can place the child in it without straining herself. At the end of several hours, hunger will wake it up, when it can be nursed, replaced in its crib and sleep soundly until the morning, if it has not been allowed to sleep too long or too late in the afternoon, and thus afford the wearied mother a delicious night's rest, to arise in the morning with a renovated system, refreshed, thankful, and hopeful, and ready to enter on the duties of the day with a light and cheerful heart. On the other hand, in consequence of bad management and a want of system as to the times of eating and sleeping for the nursling, and by keeping it in the same bed with her, it becomes restless, it wakes up a dozen times perhaps in a night, and each time, by some noise or motion rouses the mother, with the result of depriving her of that rest and repose which she so much requires, and the morning finds the body still weary, the mind discouraged and depressed, totally unfitted for the proper discharge of household duties, as is too plainly indicated by the expression of listlessness and sadness which pervades the features. Indeed, a mother can better afford to eat too little than to sleep too little, but by arranging to have the regularities named carried out for several nights in succession, there will be a happy change in all respects. When a child is six months old, it can safely fast five or six hours if asleep, and, as before, if fed a little before sun down, it should be put to bed a little later, and not be allowed to take any thing more until the mother retires for the night, which may be about ten o'clock, and if nursed then, it need not be repeated until the morning, thus allowing the mother to have her "first" sleep uninterrupted, a consummation so earnestly desired by many an overtaxed wife, but which she is unable to arrange for want of a little thought, firmness and management. The reader is earnestly requested to make particular note of it, that the seeds of a lifetime suffering, if not an early death, are sown in the constitutions of children by their own mothers during the nursing period. Millions of children die before they are two years old, by a wrong system of feeding, originating in the ignorance of the parents. The instinct and the highest pleasure of the new-born child is to eat, it is the balm for all its cries, it hushes every complaint. The young mother soon finds this out, and putting it to the breast is the panacea for infant fretfulness. But it soon happens that the stomach is overtaxed. A second feeding occurs before the first has been disposed of; the stomach is thus kept working all the time, and soon has not the strength to work any longer, and the food being unacted upon, begins to ferment, turns sour, generates wind, and this is the "colic" of infancy. Colic gives pain, pain excites crying, to quiet which, food is given, or "soothing" syrups are administered, with the inevitable result, in all cases, of exaggerating the trouble sooner or later; and in countless instances, there is a speedy and entire breaking down of the system, and death ends the outrage, as to the child, but in the mean while, by reason of the child's sufferings, many a night has been passed in sleepnessness by both parents.

